# Species and population specific gene expression in blood transcriptomes of marine turtles

**DOI:** 10.1186/s12864-021-07656-5

**Published:** 2021-05-13

**Authors:** Shreya M. Banerjee, Jamie Adkins Stoll, Camryn D. Allen, Jennifer M. Lynch, Heather S. Harris, Lauren Kenyon, Richard E. Connon, Eleanor J. Sterling, Eugenia Naro-Maciel, Kathryn McFadden, Margaret M. Lamont, James Benge, Nadia B. Fernandez, Jeffrey A. Seminoff, Scott R. Benson, Rebecca L. Lewison, Tomoharu Eguchi, Tammy M. Summers, Jessy R. Hapdei, Marc R. Rice, Summer Martin, T. Todd Jones, Peter H. Dutton, George H. Balazs, Lisa M. Komoroske

**Affiliations:** 1 Department of Environmental Conservation, University of Massachusetts, Amherst, MA USA; 2 Marine Turtle Biology and Assessment Program, Protected Species Division, Pacific Islands Fisheries Science Center, National Marine Fisheries Service, National Oceanic and Atmospheric Administration, Honolulu, HI USA; 3 Marine Mammal and Turtle Division, Southwest Fisheries Science Center, National Marine Fisheries Service, National Oceanic and Atmospheric Administration, La Jolla, CA USA; 4 Chemical Sciences Division, National Institute of Standards and Technology, Hawai’i Pacific University, Waimanalo, HI USA; 5 Department of Anatomy, Physiology and Cell Biology, University of California, Davis, Davis, CA USA; 6 Center for Biodiversity and Conservation, American Museum of Natural History, New York, NY USA; 7 New York University, New York, NY USA; 8 School of Agricultural, Forest, and Environmental Sciences, Clemson University, Clemson, SC USA; 9 United States Geological Survey, Wetland and Aquatic Research Center, Gainesville, FL USA; 10 Section of Molecular Biology, Division of Biological Sciences, University of California, San Diego, La Jolla, CA USA; 11 Marine Mammal and Turtle Division, Southwest Fisheries Science Center, National Marine Fisheries Service, National Oceanic and Atmospheric Administration, Moss Landing, CA 95039 USA; 12 Moss Landing Marine Laboratories, San Jose State University, Moss Landing, CA 95039 USA; 13 Department of Biology, San Diego State University, San Diego, CA USA; 14 Rainbow Connection Research, Guam, USA; 15 Jessy’s Tag Services, Saipan, Commonwealth of the Northern Mariana Islands USA; 16 Hawai’i Preparatory Academy, Kamuela, HI USA; 17 Golden Honu Services of Oceania, Honolulu, HI USA

**Keywords:** Comparative transcriptomics, Sea turtle, Minimally invasive sampling, Conservation physiology, RNA-sequencing, Ortholog

## Abstract

**Background:**

Transcriptomic data has demonstrated utility to advance the study of physiological diversity and organisms’ responses to environmental stressors. However, a lack of genomic resources and challenges associated with collecting high-quality RNA can limit its application for many wild populations. Minimally invasive blood sampling combined with de novo transcriptomic approaches has great potential to alleviate these barriers. Here, we advance these goals for marine turtles by generating high quality de novo blood transcriptome assemblies to characterize functional diversity and compare global transcriptional profiles between tissues, species, and foraging aggregations.

**Results:**

We generated high quality blood transcriptome assemblies for hawksbill (*Eretmochelys imbricata*)*,* loggerhead (*Caretta caretta*), green (*Chelonia mydas*), and leatherback (*Dermochelys coriacea*) turtles. The functional diversity in assembled blood transcriptomes was comparable to those from more traditionally sampled tissues. A total of 31.3% of orthogroups identified were present in all four species, representing a core set of conserved genes expressed in blood and shared across marine turtle species. We observed strong species-specific expression of these genes, as well as distinct transcriptomic profiles between green turtle foraging aggregations that inhabit areas of greater or lesser anthropogenic disturbance.

**Conclusions:**

Obtaining global gene expression data through non-lethal, minimally invasive sampling can greatly expand the applications of RNA-sequencing in protected long-lived species such as marine turtles. The distinct differences in gene expression signatures between species and foraging aggregations provide insight into the functional genomics underlying the diversity in this ancient vertebrate lineage. The transcriptomic resources generated here can be used in further studies examining the evolutionary ecology and anthropogenic impacts on marine turtles.

**Supplementary Information:**

The online version contains supplementary material available at 10.1186/s12864-021-07656-5.

## Background

Transcriptomics has become a powerful tool to study the underpinnings of ecological and physiological diversity within and between species [1]. In particular, RNA-sequencing can be used to characterize global gene expression and sequence diversity across functional components of the genome. Combined with advances in bioinformatics approaches, high-throughput sequencing has enabled the completion of studies in wild populations with limited genomic resources that were previously not possible. De novo transcriptome assemblies paired with analyses to identify orthologs derived from common ancestral genes have facilitated comparisons of functional diversity and gene expression between closely-related species, especially when reference genomes are not available [2–5]. Additionally, transcriptomics is becoming increasingly employed to complement other methods of assessing physiological responses to environmental conditions, such as hormone assays and blood biochemistry analyses [6–9]. For example, transcriptomics has been used to identify differing physiological responses in urban and rural dwelling great tits (*Parus major* [8]) and for setting baselines and identifying potential cold adaptation mechanisms in dolphins (*Tursiops truncatus* [10]) and beluga whales (*Delphinapterus leucas* [11]).

Although RNA-sequencing techniques have become more feasible in non-model systems, collecting tissues that yield high-quality RNA remains a challenge in many wild populations. This is especially true for protected or long-lived species where non-lethal, minimally-invasive sampling is necessary. Characterizing transcriptomes from blood samples is appealing because blood circulates through the whole body and perfuses most organs and other tissues. Its utility as a liquid biopsy has been developed in human and wildlife medicine [12–14]. While blood does not capture the full array of physiological functions within an organism’s tissues, blood transcriptomes have been shown to contain two thirds of orthologous genes present in liver samples (an organ with high functional gene expression diversity frequently used in transcriptomics studies) in six species of reptiles [15], and contain 61% of protein coding genes in the genome of a species of bat [16]. Additionally, reptile blood samples include both nucleated red and white blood cells, so it is possible to obtain a sufficient amount of RNA from a small volume of blood [15, 17, 18], making blood transcriptomes a valuable tool to understand functional diversity in reptiles and potentially to develop biomarkers for physiological and health assessments.

Marine turtles are reptiles of conservation concern with a growing but limited body of genomic resources [19]. This taxon is globally distributed and has some of the longest known migrations on the planet, so a single individual may experience a wide range of environmental conditions and anthropogenic impacts, which have the potential to be cumulative, within its lifetime [20]. Six out of seven extant species are listed in an elevated threat category (vulnerable, endangered, or critically endangered) on the IUCN Red List and under the U.S. Endangered Species Act [21, 22]. Marine turtles face a myriad of threats, such as fisheries interactions, intentional harvest of eggs and meat for consumption, environmental contaminants, climate change, and disease [23–27]. While there are some characteristics shared by all or multiple species of marine turtle, each species, and sometimes populations within a species, have unique ecological adaptations and life history traits. For example, the trophic ecology varies widely between hawksbill (*Eretmochelys imbricata;* primarily spongivores)*,* loggerhead (*Caretta caretta;* omnivores), green (*Chelonia mydas;* herbivores or omnivores depending on population or life stage), and leatherback (*Dermochelys coriacea;* gelatinivores) turtles [28]. Leatherback turtles also exhibit regional endothermy and other specialized physiological adaptations to inhabit cold water [29, 30]. The evolutionary divergence between Dermochelidae-Cheloniidae (the two extant marine turtle families containing the leatherback and hardshell marine turtle species, respectively) is estimated at 55–100 million years ago [31, 32], but turtles have slower rates of evolution compared to other vertebrates [33] and marine turtles can have high rates of sequence conservation between species [34]. Thus, these unique physiological and ecological adaptations may be driven largely by key functional differences within a small proportion of their total genomes. Modulating gene expression can also be a mechanism of local adaptation and a source of evolutionary novelty between populations within a species [35, 36]. Gene expression profiles vary between geographically distinct populations and can also change based on environmental conditions such as water temperatures and life stage [9]. Thus, comparative transcriptomics approaches can identify potential drivers of the observed ecological diversity between and within marine turtle species, and offer key insight into how they modulate their physiology in response to natural and anthropogenically driven environmental conditions.

Here, we present the first multi-species comparison of marine turtle transcriptomes. In this study, we assembled de novo blood transcriptomes and examined gene expression across four species of marine turtles to characterize and compare the transcriptomic diversity within and across species. We also conducted functional annotation to explore the biological processes represented in genes expressed in blood. To further assess the utility of blood transcriptomes compared to other tissues commonly used for transcriptomic studies, we quantified the proportion of genes shared between blood, brain, lung, and ovary transcriptomes for leatherback turtles. Finally, we used differential gene expression and functional gene enrichment analyses to explore potential drivers of responses to varying environmental conditions within green turtle foraging aggregations. Green turtles have a global distribution comprised of eleven distinct population segments [37] that are genetically differentiated, have different life histories, and face varying levels of anthropogenic disturbance. Here, we include samples from three populations (East Pacific, Central North Pacific, and Central West Pacific), including individuals (East Pacific) that inhabit highly urbanized estuaries. Collectively, these analyses serve to demonstrate the potential of transcriptomics studies using minimally invasive blood sampling to advance our understanding of marine turtle evolutionary ecology and conservation biology.

## Results

### Transcriptome assessment & annotation

We conducted RNA-sequencing of blood samples from green, hawksbill, leatherback, and loggerhead turtles (*n* = 43), and used these data to assemble four species-specific blood transcriptomes. We also used public data in the NCBI Sequence Read Archive to assemble leatherback tissue-specific transcriptomes. Sequencing yielded 32.7 ± 5 million raw reads per sample (mean ± standard deviation; Table S1), with an average of 5.5 ± 2.3% (mean ± standard deviation) of reads mapping to hemoglobin. Filtering to collapse transcripts with high sequence similarity and to remove redundant, low quality, or chimeric transcripts reduced the number of transcripts in assemblies by 27.9 ± 7.6 % (mean ± standard deviation) compared to raw assemblies. Transcriptomes had > 75 and 71% mapping rates for conspecific and heterospecific samples, respectively (Table 1). All filtered assemblies had BUSCO completeness scores > 72% (Table 2), and N50 > 2000. A total of 844 (0.8%) of all amino acid sequences in the green turtle filtered assembly matched to bacterial, archaeal, or viral sequences, indicating low levels of non-host contamination.

**Table 1:** Quality assessment metrics of unfiltered and filtered transcriptome assemblies for multiple tissue types collected from four marine turtle species

	Loggerhead ***- blood***		Hawksbill ***- blood***		Green turtle ***- blood***		Leatherback ***- blood***		Leatherback ***- brain***		Leatherback ***- lung***		Leatherback ***- ovary***	
	*raw*	*filtered*	*raw*	*filtered*	*raw*	*filtered*	*raw*	*filtered*	*raw*	*filtered*	*raw*	*filtered*	*raw*	*filtered*
**Total trinity transcripts**	132,146	77,392	280,711	220,458	489,355	376,736	347,717	276,709	216,942	140,332	243,118	165,611	163,840	119,574
**Contig N50**	3032	2552	3143	2276	3221	2303	2867	2187	3618	2788	3288	2526	3050	2373
**Median contig length**	675	707	574	529	606	575	597	553	666	629	632	601	673	593
**Mean mapping rates**														
*Conspecific samples*	91.50%	75.36%	95.53%	93.58%	94.88%	93.94%	95.49%	94.95%	92.98%	83.22%	92.52%	82.02%	94.96%	93.89%
*Heterospecific samples*	82.65%	69.54%	88.56%	85.44%	86.24%	85.99%	83.58%	83.14%	N/A	N/A	N/A	N/A	N/A	N/A
**Transrate scores**														
*Assembly score*	0.23	0.35	0.25	0.36	0.29	0.42	0.26	0.37	0.21	0.31	0.21	0.29	0.20	0.29
*Optimal assembly score*	0.35	0.36	0.36	0.37	0.42	0.43	0.36	0.38	0.33	0.32	0.30	0.30	0.30	0.30

**Table 2:** BUSCO completeness percentage scores based on the vertebrata database for unfiltered and filtered transcriptome assemblies for multiple tissue types collected from four marine turtle species

	Loggerhead ***- blood***		Hawksbill ***- blood***		Green turtle ***- blood***		Leatherback turtle ***- blood***		Leatherback ***- brain***		Leatherback ***- lung***		Leatherback ***- ovary***	
	*raw*	*filtered*	*raw*	*filtered*	*raw*	*filtered*	*raw*	*filtered*	*raw*	*filtered*	*raw*	*filtered*	*raw*	*filtered*
**Total Complete BUSCOs**	76.7	72.8	81.1	80.7	83.7	83.7	84.9	85	90.6	86.3	89.5	86.4	88.9	89
**Single-copy complete BUSCOs**	37.3	50.9	33.9	46.6	31.2	43.4	32.8	45.4	40.9	57.2	39.7	55.5	37.2	57.5
**Duplicated Complete BUSCOs**	39.4	21.9	47.2	34.1	52.5	40.3	52.1	39.6	49.7	29.1	49.8	30.9	51.7	31.5
**Fragmented BUSCOs**	6.3	7.1	5.5	5.6	5.4	5.5	4.5	4.2	3.1	4.1	4.1	5	3.9	3.7
**Missing BUSCOs**	17	20.1	13.4	13.7	10.9	10.8	10.6	10.8	6.3	9.6	6.4	8.6	7.2	7.3

We functionally annotated the green turtle blood transcriptome using Blast2GO to investigate the functions of genes shared or differentially expressed between species or green turtle foraging aggregations [38]. Biological processes represented in the green turtle blood transcriptome are shown in Figure S1 and Table S2. Blast2GO retrieved BLAST hits for 44.4% of transcripts, gene ontology (GO) mappings for 33.9% of transcripts, and 24.7% of transcripts were ultimately annotated with GO terms. These annotated transcripts were associated with 19,583 GO terms across all three GO domains (cellular component, molecular function, and biological process). Of the annotated GO terms in the biological process category, the majority fell within biosynthetic processes (~ 15,000), followed by cellular protein modification processes, signal transduction, cellular nitrogen compound metabolic processes, and stress response (Figure S1). Sequences in the green turtle blood transcriptome were involved with 140 KEGG (Kyoto Encyclopedia of Genes and Genomes) pathways [39]. The most complete KEGG pathways (highest number of pathway enzymes represented in transcriptome) included purine, amino sugar, glycine, glycerophospholipid, and pyrimidine metabolism. We also observed high numbers of sequences mapping to specific enzymes involved in numerous pathways. For example, 979 transcripts were annotated with enzyme code 3.1.3.16-phosphatase, which was involved in the T cell receptor signaling pathway, PD-L1 expression and PD-1 checkpoint pathway in cancer, and Th1 and Th2 cell differentiation (Table S3).

To examine the functions of genes shared between leatherback tissues and blood, we also functionally annotated a combined-tissue leatherback transcriptome. Annotation of the combined leatherback tissue transcriptome yielded BLAST hits for 63% of transcripts, GO mappings for 48. 9% of transcripts, and 48.5% of transcripts were ultimately annotated with GO terms (Figure S2 and Table S4). However, we note that the higher annotation percentages here compared to the green turtle blood transcriptome were likely due to an additional filtering step applied in our computational streamlined methods using Transdecoder (i.e., smaller input file containing only 77,387 transcripts identified as containing open reading frames). Annotated transcripts were associated with 23,859 unique GO terms across all three GO domains. Within the biological process category, the most abundant GO terms were related to signal transduction, biosynthetic process, cell differentiation, cellular protein modification, and response to stress. Annotated leatherback transcripts were involved in 149 KEGG pathways ( [39], Table S3). The most complete KEGG pathways were also all related to amino acid metabolism (e.g. purine, glycine, pyrimidine, arginine), though these differed slightly in comparison to the green turtle annotation above. We also observed high numbers of sequences mapping to specific enzymes involved in numerous pathways. For example, 680 transcripts were annotated as part of the serine/threonine protein kinase enzyme, which is involved in thermogenesis, relaxin signaling, and numerous viral infection KEGG pathways.

### Shared orthology between species and tissues

**Fig. 1:**
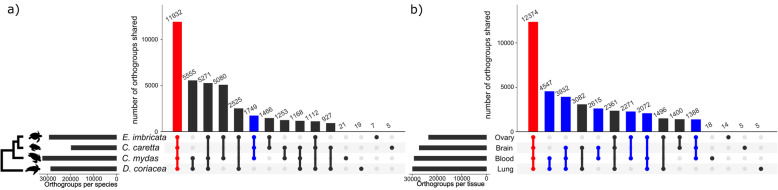
Shared and unique orthogroups between transcriptome assemblies. **a** Shared orthogroups between blood transcriptomes from four species of marine turtles, hawksbill (*E. imbricata*), loggerhead (*C. caretta*), green (*C. mydas*), and leatherback (*D. coriacea*). Red represents a “core set” of orthogroups represented in all species and blue represents orthogroups shared among all hardshell species. The cladogram on the left represents the phylogenetic relationships between these species as reported by Duchene et al. ([31]; note that branch lengths depicted are representative of relative relationships only, and not drawn to scale to represent estimated divergence times). **b** orthogroups shared between four leatherback tissues (ovary, brain, blood, and lung). Red represents orthogroups shared between all four tissues and blue represents orthogroups present in tissue combinations that include blood

**Fig. 2:**
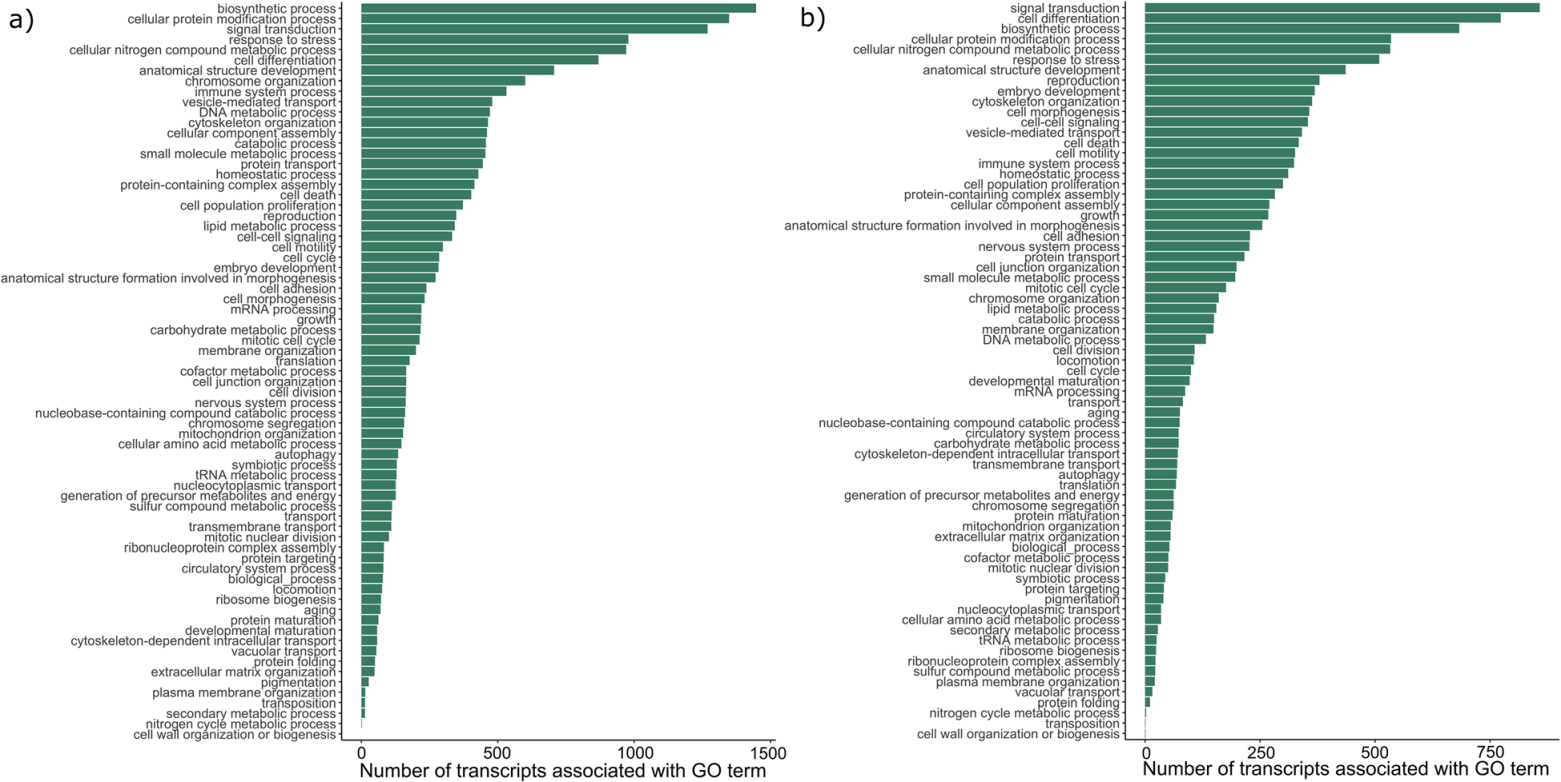
GO Slim categories in shared orthogroup sets. The number of genes in each GO slim functional category **a** from green turtle blood transcriptome genes that belonged to orthogroups present in all four species’ blood transcriptomes and **b** multi-tissue leatherback transcriptome genes that belonged to orthogroups present in all four leatherback tissues

There was a combined total of 267,039 transcripts in all four species-specific blood transcriptomes, and 64.3% of these transcripts were assigned to orthogroups (Fig. 1a; Table S5) via protein orthology analysis. A total of 11,932 orthogroups were shared between all four species-specific blood transcriptomes (31.3% of all orthogroups identified). This was the largest shared set of orthogroups, and likely represents a core set of genes expressed in blood across marine turtles. The largest functional groups of genes in this core set based off the green turtle transcriptome annotation were biosynthetic processes (*n* = 1447 genes), cellular protein modification processes (*n* = 1348 genes), and signal transduction (*n* = 1269 genes; Fig. 2a, Table S2). Additionally, this ‘marine turtle core gene set’ contained 84.4% of the genes in the core set across reptilian blood transcriptomes previously identified by Waits et al. [15]. There were few species-specific orthogroups identified (≤ 60, Fig. 1a), however, it is important to note that this is distinct from species-specific unique genes expressed because orthogroups are only assigned if more than one transcript (within or between species) is in the set [40]. The relative set size of shared orthogroups was not in complete concordance with phylogenetic distances between species. Specifically, although leatherback turtles have the greatest divergence from the other species ( [31], Fig. 1a), the number of orthogroups shared among the three hardshell species was lower than the numbers of orthogroups shared among several other groups containing hardshell species and the leatherback turtle. However, all of the groups in the latter category were missing the loggerhead, for which only a single sample was available.

In a comparison of the leatherback blood transcriptome to those of more traditionally sampled organs, 69.5% of 228,977 total transcripts were assigned to an orthogroup by protein orthology analysis (Fig. 1b and Table S6). This comparison revealed that a large proportion of identified orthogroups were expressed in all four tissues (12,374 orthogroups, 32.9% of total orthogroups identified; Fig. 1b and Table S6). The largest functional groups of genes in this core set based off the multi-tissue leatherback transcriptome annotation were signal transduction (*n* = 858 genes), biosynthetic processes (*n* = 683 genes), and cell differentiation (*n* = 773 genes; Fig. 2b, Table S4). Secondly, 44.8% of orthogroups were expressed in other combinations of tissues that included blood. Similar to blood transcriptome comparisons across species, there were few tissue-specific orthogroups (42 orthogroups, 0.11% of total orthogroups), which contained 137 transcripts (0.06% of all transcripts present in the four assemblies).

### Transcriptional signatures across species

**Fig. 3:**
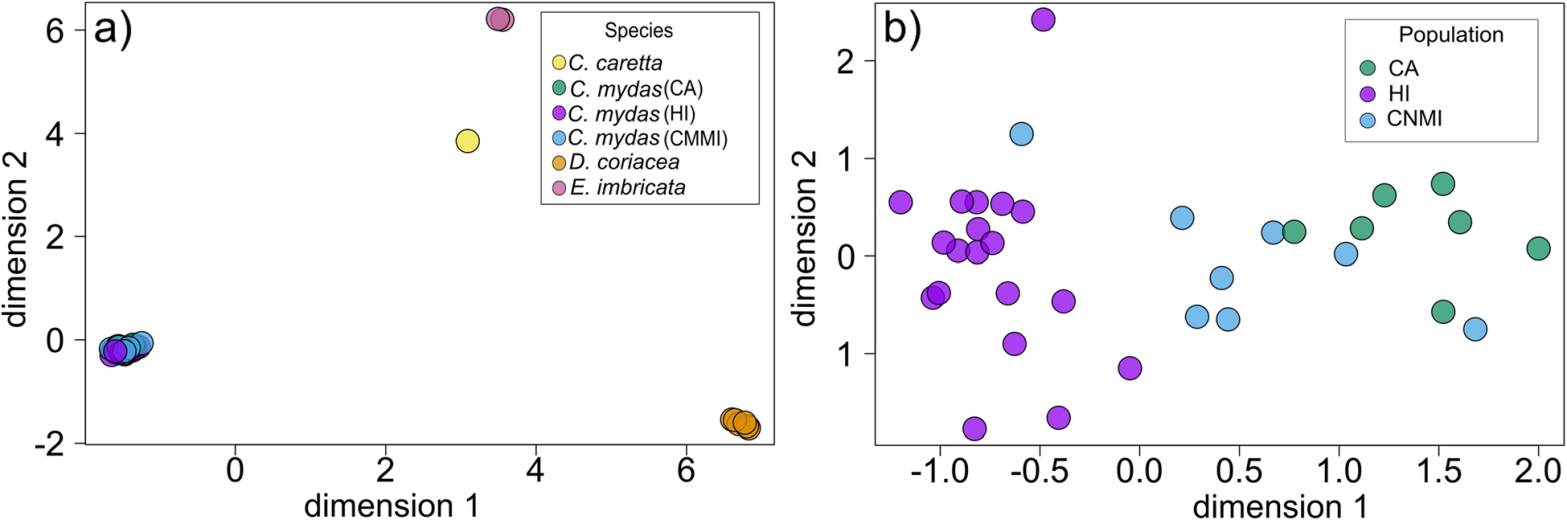
Multidimensional scaling plots of global transcriptomic signatures. **a** All species based on filtered counts at orthogroup level, and **b** green turtle foraging aggregations only based on filtered counts at gene level

Multi-dimensional scaling (MDS) revealed distinct clustering by species (Fig. 3a), indicating that transcriptional signatures of shared genes vary among species. Exploratory differential expression analysis including only orthogroups shared between the three species with more than one sample available (green turtles, hawksbills, and leatherbacks) further identified that 47.4 –57.4% of shared orthogroups were significantly different among the species (Table S7).

### Differential gene expression among green turtle foraging aggregations

**Fig. 4:**
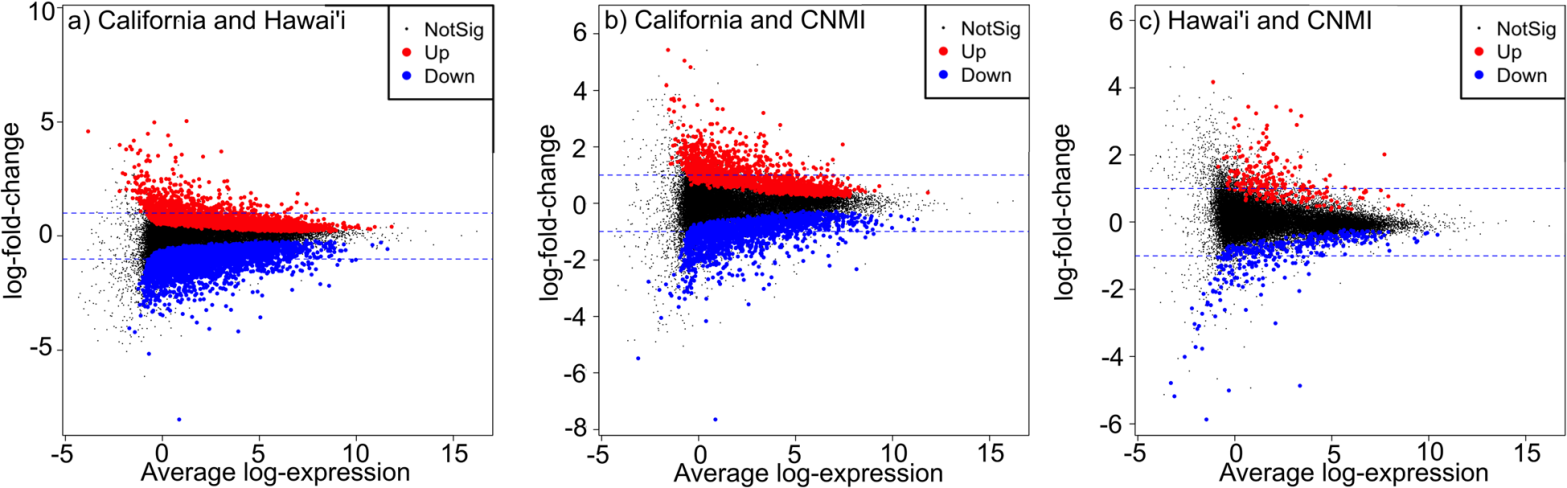
Differential gene expression between green turtle foraging aggregations. Log-fold expression changes between green turtles sampled in **a** California and Hawai’i, **b** California and the Commonwealth of the Northern Mariana Islands (CNMI), and **c** Hawai’i and the CNMI. Each dot represents one gene. Genes significantly upregulated and downregulated in respect to the first population listed in each pair are denoted in red and blue, respectively (FDR < 0.05). Dotted blue lines represent log fold change = ±1

**Fig. 5:**
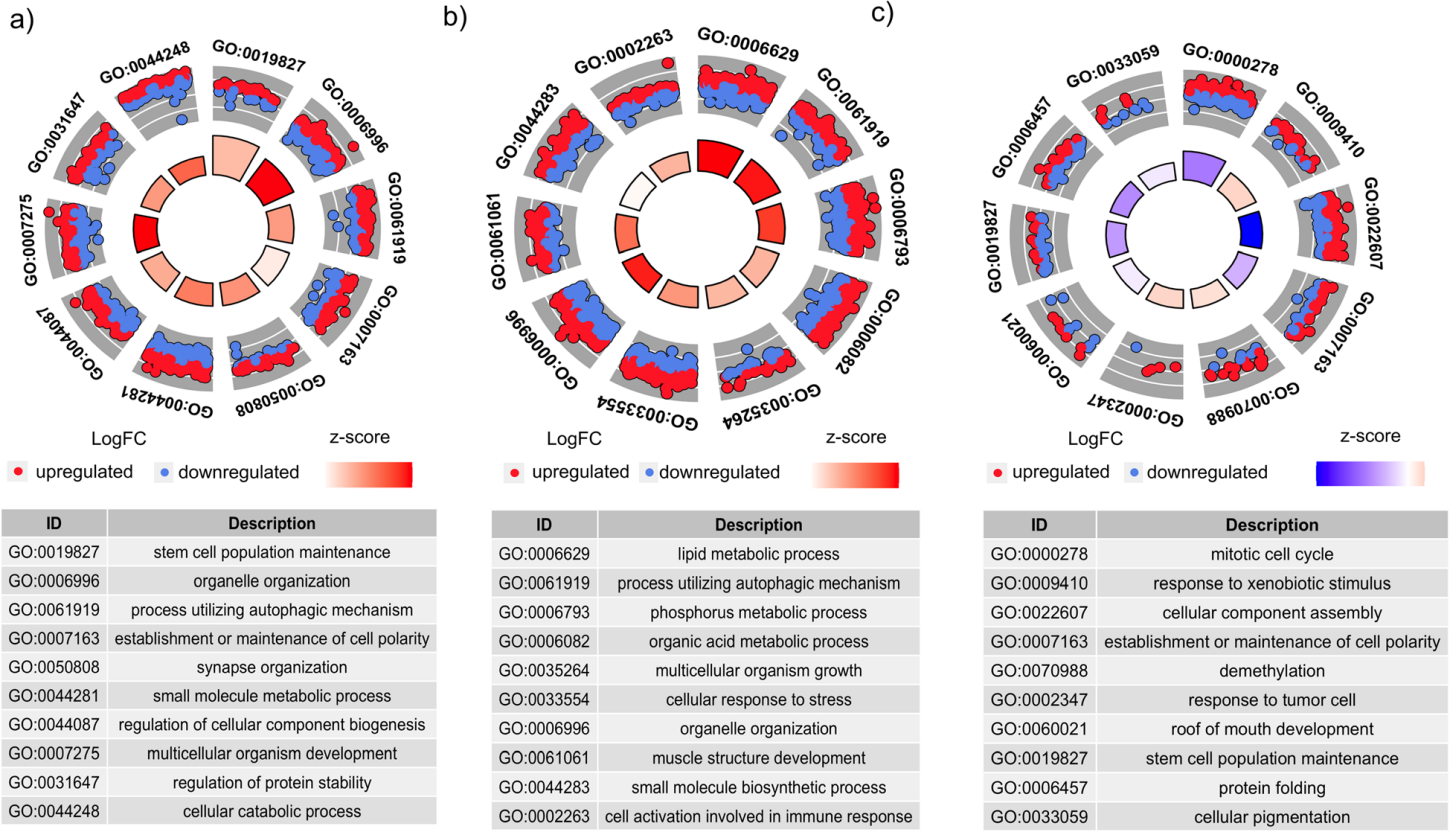
Functional enrichment analyses. GOcircle plots display scatter plots of log fold change (logFC) for the most statistically significant GO terms. Red dots represent upregulated genes and blue dots represent down regulated genes. The inner circles display z-scores calculated as the number of up-regulated genes minus the number of down-regulated genes divided by the square root of the count for **a** California and Hawai’i, **b** California and the Commonwealth of the Northern Mariana Islands (CNMI), and **c** Hawai’i and the CNMI. Up-regulated means that expression is higher in the population listed second, because the population listed first is used as the reference level of expression

Green turtle gene expression signatures in our MDS analysis clustered by foraging aggregation, but to a lesser degree than among species (Fig. 3b). We found significant differential gene expression between all three pairwise comparisons of green turtle foraging aggregations, with the most differentially expressed genes between Hawai’i and California green turtles (6649 genes, FDR < 0.05), and the least between Hawai’i and Commonwealth of the Northern Mariana Islands (CNMI) green turtles (600 genes, FDR < 0.05) (Fig. 4 and Table S8). Thirty genes were differentially expressed in all three pairwise foraging aggregation comparisons (Table S8). Biological functions of these genes included response to oxidative stress, immune response, DNA repair, and others (see annotations in Table S2). Functional enrichment analyses for each pairwise comparison revealed a total of 16 enriched GO terms at *P* < 0.01 and 78 enriched GO terms at 0.001 < *P* < 0.05 (Fig. 5, Table S9). The top three most significantly enriched GO terms represented stem cell population maintenance, organelle organization, and processes using autophagic mechanisms, all in the California and Hawai’i pairwise comparison. The top two enriched GO terms were found in all three pairwise comparisons (*P* < 0.05). Some other enriched (0.001 < *P* < 0.05) GO terms of potential interest for future biomarker development included cellular response to stress, cell activation involved in immune response, and leukocyte mediated immunity.

## Discussion

Global transcriptomics has emerged as a robust approach to understand the mechanistic underpinnings of biodiversity and organisms’ responses to environmental stressors [1, 2, 7, 8]. It is also well-suited to complement traditional physiological datasets, such as clinical blood panels and hormone assays. However, until genomic resources and techniques for high quality sample collection are available, its practical utility for isolated and endangered populations will remain limited. Here, we generated high quality de novo transcriptome assemblies for four species of marine turtles and demonstrate that blood is a promising tissue that can be collected using non-lethal and minimally invasive sampling methods for transcriptomic studies. We reported sample collection and sequencing preparation techniques that yield high quality data from marine turtle blood and provide transcriptomes which can be used by other researchers. We characterized gene expression differences at both the species and population levels, which, in future studies, can be paired with complementary data sets to investigate linkages with environmental conditions. We also identified core sets of shared and unique genes among species that may have applications in studies of marine turtle ecological and physiological diversity, as well as the development of potential biomarkers for environmental stress responses, as has been done in other wild species [41–44].

Turtle blood transcriptome assemblies from this study generally had high species-specific mapping rates, BUSCO completeness scores, and transcript diversity. Although at our depth of sequencing, some genes that were lowly expressed in blood may be omitted, overall, these metrics indicated that our blood transcriptome assemblies were robust and high quality [3, 5, 11, 45–47]. The lower mapping rate and BUSCO completeness score of the loggerhead relative to other species is likely a result of this assembly being constructed from only one individual. Notably, it also was the species missing from sets with numbers of shared orthogroups that did not align with phylogenetic distance (Fig. 2a), suggesting lower transcript diversity was likely due to shallower sequencing. Although the individual we sequenced had reasonable depth (~ 28 M reads), these results are in concordance with prior studies’ recommendations that using multiple individuals results in more complete de novo transcriptome assemblies [4] and that at least 50 M input reads are ideal for robust assemblies [5, 45]. These high-quality de novo assemblies add to the growing number of genomic resources for marine turtles and can serve as references for future gene expression or functional gene sequence analyses studies.

Using blood for transcriptomics analyses from minimally invasive non-lethal sampling can substantially expand the species and life stages from which transcriptomic data can be gathered, which is particularly important for protected species like marine turtles. Additionally, because ample high-quality RNA can be extracted from a very small volume of blood samples in reptiles [14], transcriptomic data from blood can be gathered from individuals repeatedly as part of long-term monitoring of their health and used to answer a host of ecological and evolutionary questions. However, we recognize there are limitations in using blood for transcriptomic studies, as tissue-specific expression is common [48, 49]. Nonetheless, our findings that the functional diversity in global gene expression and BUSCO completeness scores in leatherback blood was similar to three organs traditionally used in transcriptomic analyses confirm that blood has high potential in yielding informative gene expression data in marine turtles. Our orthologous protein analysis also demonstrated that the loss of represented genes is relatively modest using blood samples instead of traditionally used tissues, and is similar to what has been demonstrated in other reptile and mammal species [15, 16, 50]. Another possible concern when using blood for RNA-sequencing studies is the high hemoglobin content consuming a large proportion of sequencing effort, making it cost-inefficient without hemoglobin depletion methods [51] that are challenging in non-model species. However, we found low alignment rates to publicly available green turtle hemoglobin sequences in our samples. This was consistent across samples, distinct population segments, and species, and considerably lower than has been previously reported in other reptiles [15]. Combined with the high diversity of functional genes in blood transcriptomes, the rapidly declining costs of high-throughput sequencing, and the risk of introducing bias with more sample processing, our results suggest the most effective approach in marine turtles may be to ‘sequence through’ over-representation of hemoglobin instead of investing in taxon-specific hemoglobin depletion methods. Lastly, contamination of transcriptomes can be a concern when employing de novo assembly methods, particularly when using tissues that can harbor pathogens and parasites such as blood. However, we found very low levels of microbial contamination (0.8%) in our assemblies suggesting that microbial contamination may not be a large issue for marine turtles and can be mitigated by including a filtering step during bioinformatics analyses if needed [15]. Further, for studies that wish to characterize both host and pathogen gene expression (i.e., dual RNA-Seq [52, 53]), blood may actually offer additional advantages as a tissue of choice in this regard. Interestingly, some of the top BLAST hits for species within bacteria, archaea, and viruses from the green turtle assembly indicated the presence of pathogenic microbial species (e.g. *Acinetobacter baumannii*). Similar analyses of transcriptomic data have identified parasites in lemurs (*Indri indri* and *Propithecus diadema)* and poison frogs (Dendrobatidae) [54, 55]. By detecting pathogen RNA in blood samples, one would be able to confirm that the pathogen was alive at the time of sampling [53]. Thus, in addition to using transcriptomic data to study immune genes and identify signatures of adaptive evolution in host species alone [56], these data can be paired with pathogen screening or cultures of host blood to evaluate bacteremia or septicemia to address a diversity of complex disease ecology and co-evolutionary research questions. Overall, our findings support that blood is an excellent tissue for a minimally invasive and non-lethal liquid biopsy for marine turtle species.

The orthogroups shared between hawksbill, loggerhead, green, and leatherback blood assemblies likely represent a core set of functional genes expressed in the blood of marine turtles, though future studies including the other three extant species of marine turtles will need to confirm this finding. Moreover, the large percentage of genes shared between this core set of marine turtle blood genes and the core set of reptilian blood genes identified in Waits et al. [15] reveals that many physiological pathways are likely conserved at broader taxonomic scales, and may be useful targets for studies developing biomarkers or investigating functional diversity across Reptilia. In particular, the marine turtle core blood gene set included 138 genes from all a priori candidate groups defined by Waits et al. ( [15]; following McGaugh et al. [57]) that are of high interest for molecular evolution and functional ecology studies and biomarker development, including the mitochondrial electron transport chain, stress response, oxidative stress, and insulin-signaling pathway genes. Conversely, although we found relatively few species-specific orthogroups, this is likely not an accurate depiction of species-specific expression because Orthofinder only assigns transcripts with at least two orthologous transcripts, within or among species, to orthogroups [40], and our assemblies were constructed with variable sequencing depths among species. It is therefore likely that single copy species-specific expressed genes are underestimated in our analyses. Although it was outside the scope of aims for this study, future analyses can examine the transcripts not assigned to orthogroups to explore genes that may only be expressed in one species. Finally, within the shared orthologous genes, we observed strong differences in expression levels between species, highlighting the potential role of gene expression regulation in underlying physiological and ecological differences among species. Further studies with larger sample sizes that assess both expression regulation and sequence divergence are needed to confirm these expression differences and understand these mechanisms. While our sample sizes for inter-specific gene expression analyses were small, our data offer exciting preliminary findings that can inform future work.

Within green turtles, we found population-specific clustering and differential expression of many genes among all three foraging locations, California, Hawai’i, and the CNMI. These groups are largely demographically isolated [37], and also inhabit areas that strongly differ in habitat and anthropogenic impacts. As such, the observed location-based differences could be caused by genetic divergence related to neutral evolutionary processes (e.g., drift following reproductive isolation), different physiological responses based on environment, or most likely, a combination of both genetic and environmental influences. Population-specific gene expression has been documented in a diversity of other marine taxa, such as stony corals, teleost fishes, and intertidal copepods [58–60]. While experimental work using traditional approaches such as common garden experiments is challenging in protected species, our data can inform hypotheses about what is driving these differences [1, 61] that can be further assessed with candidate gene profiling linked to complementary datasets. To understand environmental influences, transcriptomic data can also be paired with contaminant analysis to identify correlations between gene expression and environmental pollution [62–64]. Additionally, environmental degradation has been associated with fibropapillomatosis, a tumor-forming disease of marine turtles, within the Hawai’ian Islands and globally [65–68]. Thus, identifying gene expression profiles associated with pollution could provide insight into disease emergence and aid in developing biomarkers of disease. The stronger differential expression between both insular foraging aggregations (i.e., Hawai’i and CNMI) and the California foraging aggregation is suggestive of environmental drivers because although California and Hawai’i populations originate from the same evolutionary clade that is distinct from the CNMI population [69], the California aggregation forages in a much more highly urbanized temperate environment [70] compared to the tropical, less impacted foraging grounds of both island aggregations. Further, the Hawai’i aggregation largely forages and nests within the greater Hawai’ian islands [71, 72], so there is likely limited contemporary gene flow among the Hawai’ian and CNMI populations. Finally, GO terms representing cellular response to stress and cell activation involved in immune response were significantly enriched in comparisons between California and both island foraging aggregations, and leukocyte-mediated immunity was also enriched between the California and Hawai’i foraging aggregations. Thus, although we cannot draw causative conclusions from our current dataset, it is a plausible hypothesis that differential expression of genes in these functional groups may be in response to differences in exposure to pollution and other stressors in urbanized versus insular locations. Relationships between environmental stressors such as pollutant and pathogen exposure and transcriptomic responses have been documented in a wide variety of taxa, including great tits [8], killifish (*Fundulus heteroclitus* [73]), and wild salmon smolts (*Oncorhynchus nerka* [74]). Additionally, correlations between stress hormones and gene expression have been documented in elephant seals (*Mirounga angustirostris* [75]). Transcriptomic data can also be paired with sex or life stage information to explore the potential for developing gene expression-based biomarkers to determine important demographic information such as sex, which would be a useful addition to the current methods available for sexing immature marine turtles [76, 77]. Thus, future studies pairing gene expression data with contaminant profiles, disease status, environmental data, and health assessment biomarkers, while factoring baseline expression differences between groups demonstrated in this study, can strengthen our understanding of potential relationships between differential gene expression and environmental stress in wild populations.

## Conclusions

Minimally invasive blood sampling combined with de novo transcriptomic approaches has strong potential to alleviate key barriers of applying transcriptomic tools in wild, protected populations. Our study provides genomic resources for non-model species of high interest for conservation and demonstrates how global gene expression data from blood can be used to explore evolutionary ecology and anthropogenic impacts on marine turtles and other species where traditional lethal sampling is unwarranted. The distinct differences in gene expression signatures between species and populations yield insight into the functional genomics underlying the diversity in this ancient vertebrate lineage, and the high-quality transcriptomes and expression analyses provide key baseline information to inform a variety of future transcriptomic applications in marine turtles.

## Methods

### Sample collection and RNA extraction

Blood samples were collected between 2012 and 2016 from hawksbill turtles from off Palmyra Atoll (U.S. Minor Outlying Islands) and the CMNI (*n* = 2), a loggerhead off the southern California coast (*n* = 1), leatherback turtles off the central California coast (USA, *n* = 6), and from three foraging aggregations of green turtles in Southern California (USA, *n* = 7, East Pacific population), Hawai’ian Islands (USA, *n* = 19, Central North Pacific population), and the CNMI (*n* = 8, Central West Pacific population). See Table S1 for specific sampling locations and additional details. Blood was collected from the dorsal cervical sinus using 21-gauge 3.8-cm needles for hardshell turtles [78] and 18-gauge 8.75 cm spinal needles flushed with sodium heparin for leatherback turtles, connected to a vacutainer adapter to directly fill PAXgene™ Blood tubes (Qiagen, Valencia, CA, USA) to stabilize RNA. Blood-filled PAXgene tubes were initially stored at − 20 °C followed by − 80 °C within 48 h until analysis according to manufacturer’s instructions. Samples (*n* = 29) obtained from the National Institute of Standards and Technology (NIST) Biological and Environmental Monitoring and Archival of Sea Turtle Tissue (BEMAST) cryogenics biorepository project were stored at liquid nitrogen vapor temperature.

Total RNA isolation using Qiagen’s PAXgene kits developed for mammalian samples was previously determined to be unable to yield high-quality RNA from samples with nucleated red blood cells, likely due to higher nuclease enzymes and protein content (L. Komoroske, unpublished data). Therefore, we developed a method optimized specifically for total RNA extraction from marine turtle whole blood [18], modified from Chiari and Galtier [17]. RNA quality (RNA integrity number (RIN)) and quantity were determined on a Fragment Analyzer (model number: 5200, Agilent Technologies, Santa Clara, CA) using a standard sensitivity RNA kit (Agilent Technologies, Santa Clara, CA, DNF-471). Total RNA was isolated from blood samples within 1–4 years after collection. Samples with RIN > 7.5 were used for further analysis.

### Library preparation and sequencing

We isolated mRNA from Total RNA using the NEBNext Poly(A) mRNA Magnetic Isolation Module (New England Biolabs, Ipswich, MA) followed by library preparation using the NEBNext Ultra Directional RNA Library Prep Kit for Illumina and the NEBNext Mulitplex Oligos for Illumina (New England Biolabs, Ipswich, MA) for dual indexing with modifications for half reactions [79]. Individual sample libraries were pooled in equimolar quantities, and the pooled library was sequenced in 150 bp paired-end reads across three lanes of an Illumina HiSeq 4000 (Illumina, San Diego, CA) by Novogene Corporation (Sacramento, CA). To compare blood to tissues more traditionally used for transcriptomic analyses, we used RNA-Seq data for leatherback ovary, lung, and brain tissue available in the NCBI Sequence Read Archive (SRA accession numbers: SRX8787566, SRX8787565, SRX8787564). See Availability of Data and Materials section for further information and the location of all scripts used for the analyses described below.

### Transcriptome assembly, filtering and mapping

Sequences were demultiplexed and concatenated across lanes by sample, followed by trimming for adaptor content with scythe [80] and quality with sickle (minimum Phred quality score of 20 [81]). We assembled de novo transcriptomes for each of the four species to capture species-specific transcripts and avoid mapping biases towards the two species (i.e. green and leatherback) with draft reference genomes available. We tested multiple numbers of samples to use as input for our de novo transcriptomes (*n* = 34, *n* = 19, and *n* = 4) to determine the optimal threshold of individuals for maximizing transcriptome completeness while minimizing computational demands, chimeric sequences, and false-splitting due to sequence divergence between populations [82], and concatenated reads from four Hawai’ian green turtle individuals to generate the green turtle transcriptome. Exploratory mapping of green turtle sequences to the green turtle reference genome [83] also determined that Hawai’ian green turtles expressed the highest sequence diversity, further supporting this sample selection for the de novo transcriptome assembly. We then similarly assembled species-specific blood transcriptomes for leatherbacks (*n* = 3), hawksbills (*n* = 2), and a loggerhead (*n* = 1), as well as tissue specific assemblies for leatherback ovary, brain, and lung (*n* = 1 per tissue). We also included leatherback brain, ovary, and lung in a single assembly in order to annotate the maximum number of functional genes. We used Trinity to assemble each transcriptome with in silico read normalization included to increase computational speed, and we set minimum contig length = 300 bp to minimize fragmented transcripts (v.2.85) [84]. We then filtered the assemblies to remove redundant and low quality or chimeric transcripts using TransRate (v.1.0.3 [85];) to retain only contigs that were most likely to be structurally complete and correct, and then CD-HIT-EST (v.4.8.1) [86] to collapse transcripts with greater than 95% similarity. We then used Salmon (v.1.1.0) [87] to quasi-map reads for each individual to their species-specific assembly and quantify transcript expression, followed by a modification of the gather-counts.py script [88] to convert raw counts from the salmon output into a count format compatible with edgeR [89].

### Transcriptome evaluation & sequencing efficiency

We evaluated assemblies before and after filtering using a combined suite of metrics including the N50 values and median contig lengths reported by the TrinityStats.pl script from the Trinity assembler [84], BUSCO completeness scores [90], TransRate scores [85], and mapping rates of conspecific and heterospecific reads to each assembly using Salmon [87] as described above. We also estimated the percentage of potential contaminant proteins in the green turtle assembly using the Diamond protein sequence aligner [91] against the NCBI nr database, filtered to only include top hits to species within bacteria archaea, and viruses (NCBI taxon ids: 2, 2157, 10,239). We considered sequences to be of non-host origin if blast sequence similarity was greater than 90%. Finally, high hemoglobin expression in blood has previously been shown to hinder effective RNA-sequencing in blood of some species but not others [92, 93]. However, alternative approaches to ‘sequencing-through’ this problem such as hemoglobin depletion requires custom bait design for non-model species, which incurs additional costs and adds another step of sample manipulation that may interject bias into expression profiles [51]. Thus, to assess this issue for marine turtles, we calculated the percentage of reads that aligned to five green turtle (*Chelonia mydas)* hemoglobin sequences available in the NCBI gene database (LOC102939173, LOC102945818, LOC102938944, LOC102946728, LOC102945589, NCBI, Accessed 28 April 2020) using bowtie2 (v. 2.3.4.3 [94]).

### Protein orthology between transcriptomes

To enable estimation of the proportion of shared versus unique genes between species, we first determined orthologous transcripts across our species-specific transcriptomes. This approach avoids biases that can arise when mapping multiple species to one reference transcriptome due to sequence divergence between species [4]. We translated and predicted coding regions for each of our assemblies using Transdecoder (v.5.5.0) [95] and then employed Orthofinder with default parameters to group transcripts from our species-specific assemblies into orthogroups, which are defined as sets of genes descended from a single gene in the most recent common ancestor within species groups (v.2.3.3) [40]. Using this method, only transcripts that have at least one orthologous transcript in any transcriptome are assigned to an orthogroup, so transcripts without any orthologs (within or across transcriptomes) are not retained [55]. We quantified the proportion of shared orthogroups between species in R (v3.6.3) [96] and visualized the results with the package ‘UpSetR’ [97]. After identifying a core set of orthogroups shared across all species in our study, we also compared to those in the core set of reptilian genes expressed in red blood cells previously identified by Waits et al. [15] using the Diamond protein sequence aligner [91] to identify Uniprot gene names matching those in our core set of orthogroups based on our green turtle annotation (see below). Finally, to evaluate the proportion of the exome present in blood transcriptomes, we followed the same procedure to identify and estimate shared and unique orthologs in leatherback turtles between blood and tissues with known high gene expression diversity traditionally employed in transcriptomic studies (brain, lung, and ovary).

### Functional annotation

We used Blast2GO (v.5.2.5) [38, 98] to functionally annotate the de novo green turtle transcriptome, and linked annotations to the other species-specific transcriptomes via orthogroups identified as described above. We chose to use this approach instead of annotating each transcriptome separately in order to relate the functional processes of orthogroups across species, and because we were largely focusing on differential expression and functional enrichment analyses between foraging aggregations of green turtles. Additionally, we conducted pilot analyses to confirm our expectation that species-specific transcriptome annotations would have a high concordance of gene identities within each orthogroup because each annotation is based on homologous genes in other vertebrate taxa using the same databases (all pairwise comparisons showed > 95% concordance). In brief, sequences in the final filtered green turtle assembly were compared to protein sequences in the NCBI non-redundant protein database (version 5) filtered to include only hits within vertebrata (NCBI taxonomy ID 7742) using the BLASTX-fast algorithm (e-value = 1.0e^− 3^, word size = 6, and otherwise default parameter selections [38]). BLAST hits were then mapped to GO terms (GO database accessed 11/2019), followed by annotation of GO terms to sequences (annotation cut-off threshold = 60, E-value = 1e^− 6^, otherwise default). GO annotations were confirmed and augmented using EggNOG mapping (version 2) to clusters of orthologous groups (version 5.0) [99]. Transcripts were also mapped to enzyme codes using the KEGG pathway analysis module, from which we generated KEGG pathway maps and statistics. Finally, we employed GO-Slim to reduce the specificity of GO terms assigned to sequences to yield a final set of broader functional summary statistics. To explore the identity and biological functions of shared and unique genes between blood and other tissues, we also annotated a combined assembly of all three leatherback tissues (brain, lung, and ovary). This followed the same procedure with the exception of two adjustments for computational streamlining informed by the results of the green turtle annotation: filtering with Transdecoder (5.5.0) [95] to retain only predicted coding regions prior to annotation, and blasting sequences against the NCBI non-redundant protein database (version 5) filtered to include only hits within tetrapoda (NCBI taxonomy ID 32523).

### Gene expression analyses between species

To compare gene expression between species, we condensed transcript counts to orthogroup level counts. Transcripts not assigned to an orthogroup were also excluded. We then filtered orthogroup level counts so that only orthogroups with at least one count per million (cpm) in at least two individuals (out of all individuals included) were retained and then normalized raw orthogroup level counts by library size using edgeR’s ‘TMM’ method [89]. We included all remaining orthogroups in a multi-dimensional scaling plot to visualize how orthogroup expression signatures differed between species. We conducted differential expression analysis to identify genes that may be candidates for driving species-specific signatures, although we recognize that this analysis is exploratory given our limited sample sizes and other challenges of comparing expression between divergent wild non-model species (e.g., establishing control groups is not possible and individuals may be captured in varied circumstances [4]). We excluded loggerheads from differential expression analyses because we only had data from one individual. To reduce issues of unequal sample sizes between groups, we randomly selected three green (one from each population) and three leatherback individuals to include with the two hawksbills. We included only orthogroups present in all three species to reduce biases driven by strong signals from species-specific orthogroups. We then filtered orthogroup level counts so that only orthogroups with at least one count per million (cpm) in at least two individuals (out of all individuals included) were retained, normalized raw counts by library size using edgeR’s ‘TMM’ method, and conducted differential expression analyses using the R packages edgeR and limma [89, 100].

### Differential expression & functional enrichment between green turtle foraging aggregations

To compare gene expression between green turtle foraging aggregations we condensed transcript counts to gene level counts, filtered counts to retain only genes with at least one cpm in at least seven individuals (the smallest group size), and normalized raw counts by library size using edgeR’s ‘TMM’ method [89]. We conducted multi-dimensional scaling visualization and differential expression analyses between the three green turtle foraging aggregations using the R packages edgeR and limma [89, 100]. We then performed functional enrichment analyses of pairwise comparisons between green turtle foraging aggregations with a Kolmogorov-Smirnov test (weight01 algorithm) implemented in the R package TopGO [101]. We report raw *p*-values rather than reporting p-values adjusted for multiple testing correction, and do not attribute statistical significance to an α = 0.05 threshold, because authors of TopGO caution that adjusted p-values may be misleading because p-values for each GO term are not calculated independently of other GO terms [102]. Input GO terms derived from the green turtle transcriptome annotation were filtered to biological process GO terms ≤ level 5. The median number of transcripts per GO term was 17, so we included up to 50 transcripts per GO term. Transcript level GO annotations were combined to gene level and redundant terms were removed. The top ten most enriched GO terms for each pairwise analysis were visualized with GOCircle plots using the R package GOplot [103]. GOCircle plots show scatterplots of log fold change values and z-scores (calculated as the number of up-regulated genes minus the number of down-regulated genes divided by the square root of the count) for genes that belong to the top ten most significantly enriched GO terms [103]. It is important to note that the z-score does not give any information about how significant GO terms are as highly significant GO terms can have z-scores close to zero [103].

## Supplementary Information

**Additional file 1: Table S1.** Sample metadata. Detailed metadata, raw read count, and the percent of reads that map to green (*Chelonia mydas)* turtle hemoglobin genes for each individual (*n* = 43) included in this study. Individuals without straight carapace length (SCL) denoted were not measured for these parameters.

**Additional file 2: Table S2.** Annotation and orthogroup information for green turtle blood transcriptome assembly transcripts. Transcripts that did not match to a Gene Ontology (GO) ID are not included.

**Additional file 3: Table S3.** KEGG pathway analyses results. KEGG pathways for the green turtle transcriptome and the leatherback multi-tissue assembly.

**Additional file 4: Table S4.** Annotation and orthogroup information for leatherback multi-tissue transcriptome assembly transcripts. Transcripts that did not match to a Gene Ontology (GO) ID are not included.

**Additional file 5: Table S5.** Species-specific orthogroups. Orthogroups and the transcripts that belong to each orthogroup from each species-specific blood transcriptome.

**Additional file 6: Table S6.** Tissue-specific orthogroups. Orthogroups and the transcripts that belong to each orthogroup from each individual tissue-type leatherback transcriptome.

**Additional file 7: Table S7.** Differential orthogroup expression analyses results for comparisons between marine turtle species. Log fold change and adjusted *p*-values are listed for the green v. leatherback, green v. hawksbill, and leatherback v. hawksbill turtle comparisons.

**Additional file 8: Table S8.** Differential gene expression analyses results for comparisons between green turtle foraging aggregations. Log fold change and adjusted p-values are listed for the California and Hawai’i, California and the Commonwealth of the Northern Mariana Islands (CNMI), and Hawai’i and CNMI Islands comparisons.

**Additional file 9: Table S9.** Functional enrichment analysis results. Functional enrichment analysis results for contrasts between green turtle foraging aggregations.

**Additional file 10: Figure S1.** Green turtle GO slim plots. Bar plots representing the number of genes in each Gene Ontology (GO) slim functional category from the green turtle blood transcriptome.

**Additional file 11: Figure S2.** Leatherback GO slim plots. Bar plots representing the number of genes in each Gene Ontology (GO) slim functional category from the multi-tissue (brain, lung, and ovary) leatherback turtle transcriptome.

## Data Availability

All raw RNA-seq reads are archived under NCBI BioProject # PRJNA660024 (https://www.ncbi.nlm.nih.gov/bioproject/?term=PRJNA660024) and SRA accession numbers: SRX8787564 (https://www.ncbi.nlm.nih.gov/sra/SRX8787564[accn]), SRX8787565 (https://www.ncbi.nlm.nih.gov/sra/SRX8787565[accn]), and SRX8787566 (https://www.ncbi.nlm.nih.gov/sra/SRX8787566[accn]). Annotation information is available in Supplementary Tables S2 and S4. All scripts used for data analysis as well as de novo transcriptomes assembled for this study are available at https://github.com/lkomoro/Marine-Turtle-Blood-Transcriptomes.
