# Supplementary figures and images for: Species and population specific gene expression in blood transcriptomes of marine turtles
Source: BMC Genomics. 2021 May 13;22:346. doi: 10.1186/s12864-021-07656-5 (PMC8117300; doi:10.1186/s12864-021-07656-5)

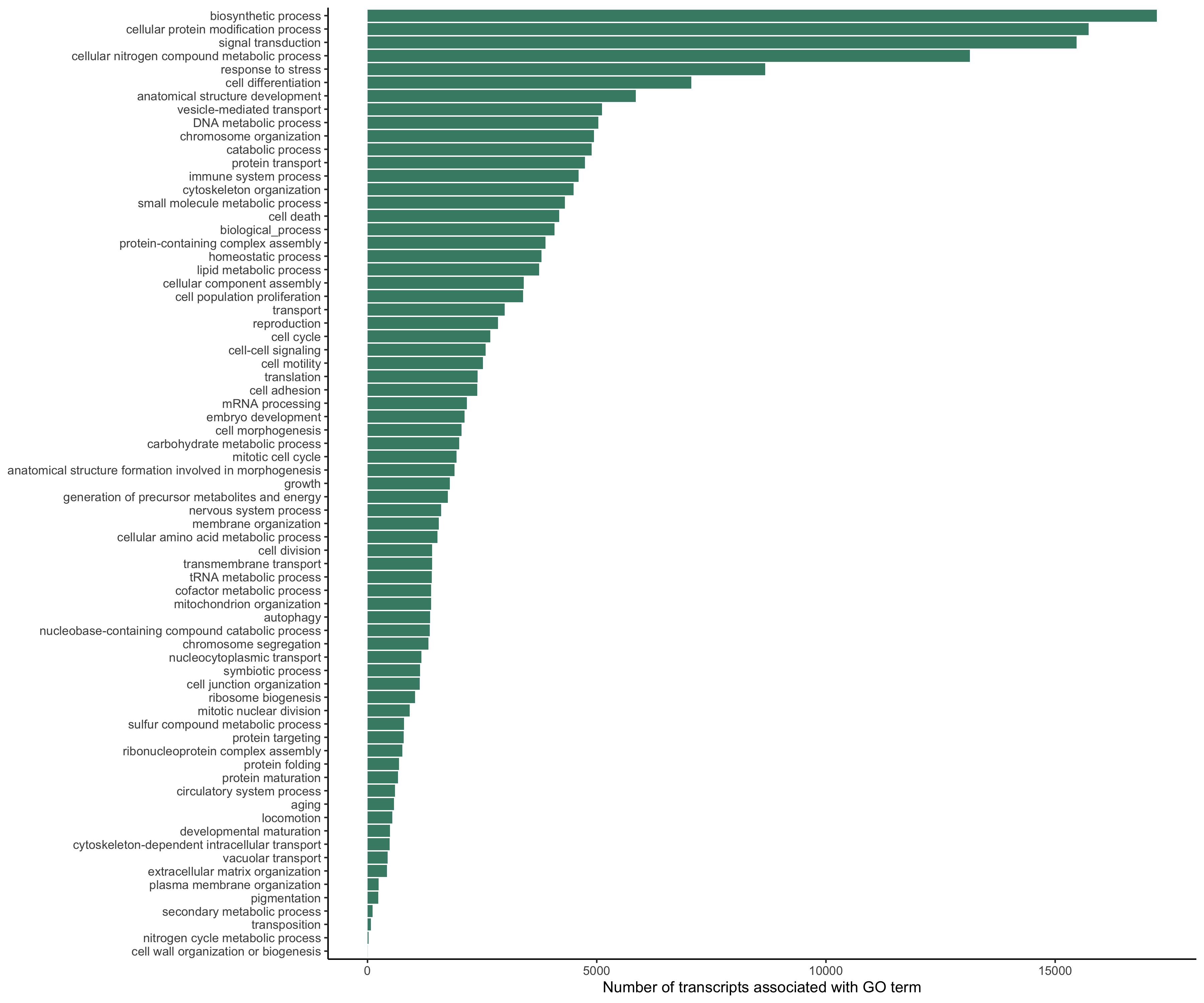

Supplement: Supplementary file 10 — Additional file 10: Figure S1. Green turtle GO slim plots. Bar plots representing the number of genes in each Gene Ontology (GO) slim functional category from the green turtle blood transcriptome. [file 12864_2021_7656_MOESM10_ESM.png]

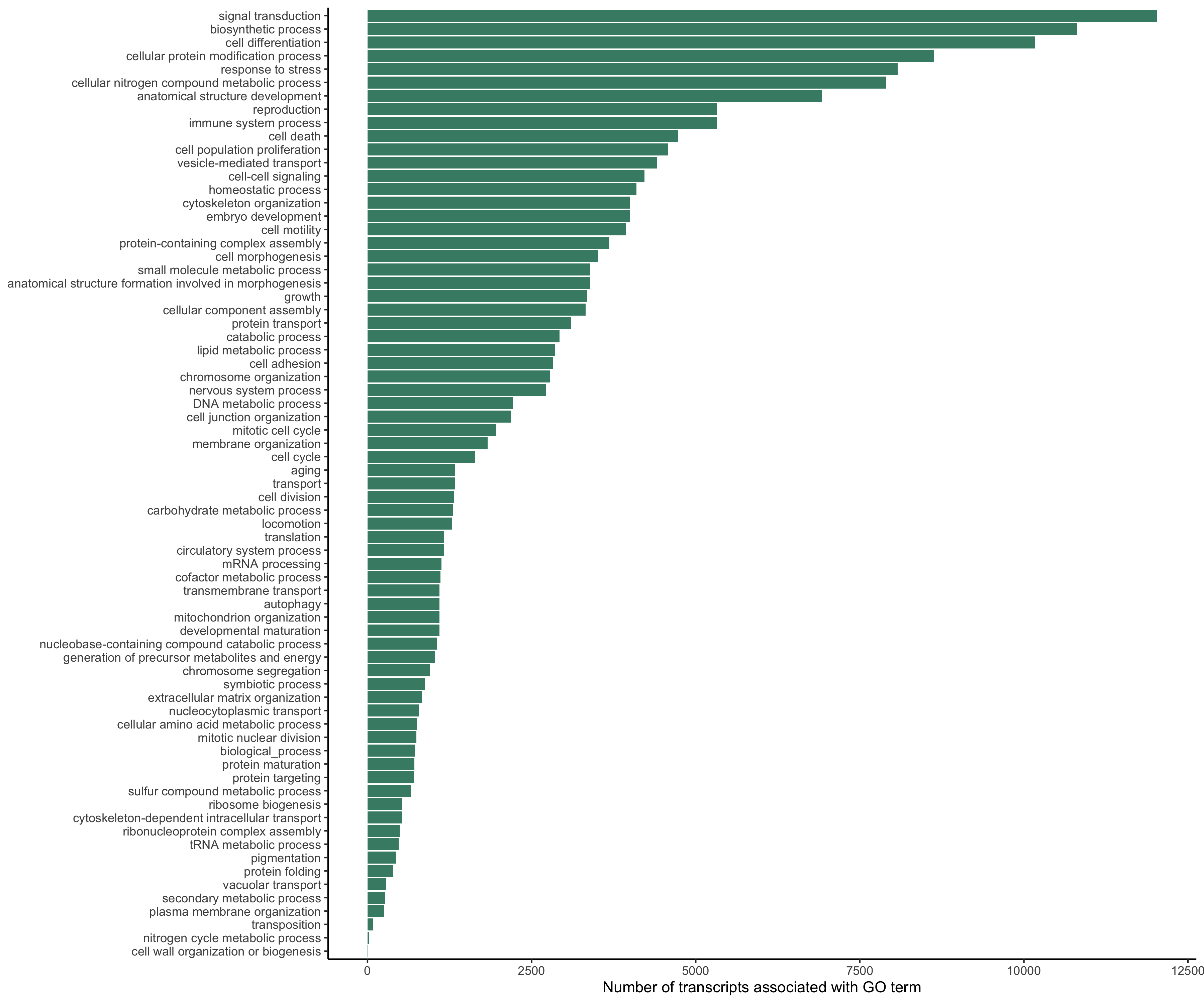

Supplement: Supplementary file 11 — Additional file 11: Figure S2. Leatherback GO slim plots. Bar plots representing the number of genes in each Gene Ontology (GO) slim functional category from the multi-tissue (brain, lung, and ovary) leatherback turtle transcriptome. [file 12864_2021_7656_MOESM11_ESM.png]
